# Using Human iPSC-Derived Neurons to Uncover Activity-Dependent Non-Coding RNAs

**DOI:** 10.3390/genes8120401

**Published:** 2017-12-20

**Authors:** Mainá Bitar, Stefanie Kuiper, Elizabeth O’Brien, Guy Barry

**Affiliations:** 1 QIMR Berghofer Medical Research Institute, Herston, QLD 4006, Australia; Maina.Bitar@qimrberghofer.edu.au (M.B.); stefanie.kuiper@griffithuni.edu.au (S.K.); Elizabeth.O’Brien@qimrberghofer.edu.au (E.O.); 2 The School of Medicine, The University of Queensland, St Lucia, QLD 4072, Australia

**Keywords:** iPSC, stem cells, neurons, non-coding RNA, neuronal activation, human-specific, RNA-Seq

## Abstract

Humans are arguably the most complex organisms present on Earth with their ability to imagine, create, and problem solve. As underlying mechanisms enabling these capacities reside in the brain, it is not surprising that the brain has undergone an extraordinary increase in size and complexity within the last few million years. Human induced pluripotent stem cells (hiPSCs) can be differentiated into many cell types that were virtually inaccessible historically, such as neurons. Here, we used hiPSC-derived neurons to investigate the cellular response to activation at the transcript level. Neuronal activation was performed with potassium chloride (KCl) and its effects were assessed by RNA sequencing. Our results revealed the involvement of long non-coding RNAs and human-specific genetic variants in response to neuronal activation and help validate hiPSCs as a valuable resource for the study of human neuronal networks. In summary, we find that genes affected by KCl-triggered activation are implicated in pathways that drive cell proliferation, differentiation, and the emergence of specialized morphological features. Interestingly, non-coding RNAs of various classes are amongst the most highly expressed genes in activated hiPSC-derived neurons, thus suggesting these play crucial roles in neural pathways and may significantly contribute to the unique functioning of the human brain.

## 1. Introduction

The brain underwent a very significant increase in size when humanoids diverged from non-human primates, with parts of the human brain being three times larger than its analogue in the chimpanzee brain [1]. This size increase was driven by the expansion of the most recently evolved brain structure, the neocortex, which is exclusive to complex primates [2]. Additionally, humans have the highest level of cortical folding (i.e., gyrification) amongst all mammals [3]. Although these and other features characterize the human brain as a unique and fascinating structure, our understanding of its molecular subtleties is still very limited.

At the genomic level, the number of protein-coding genes has remained strikingly constant throughout primates, with humans differing by only ~1% at the nucleotide level from our closest related species, the chimpanzee [4]. On the other hand, it has been observed that the number of non-coding RNAs (ncRNAs) has dramatically increased, to a point where the human genome contains at least as many ncRNA genes as protein-coding genes [5], most of which are not conserved beyond primates [6,7]. Also, further investigation showed that the number of ncRNAs correlate with organism complexity, indicating a possible link between these two observations [8,9]. Long non-coding RNAs (lncRNAs) are defined as ncRNAs longer than 200 nucleotides without protein-coding capacity and have been implicated in many roles, which include primate brain development and neuronal function [10,11]. According to the NONCODE project [5], lncRNAs can be classified according to their relative localization as antisense (~15%), intergenic (~40%), sense exonic (~25%), and sense non-exonic (~20%). A comparison between human, chimpanzee, and macaque brain transcriptomes revealed that intergenic transcripts were the most variable across species and overall differences increased with evolutionary distance [12]. Functionally, many of these intergenic RNAs seem to regulate gene expression by binding to chromatin modification complexes [13]. Furthermore, an initial study found over 800 antisense transcripts to naturally occur in the human transcriptome, suggesting an important role for such lncRNAs, which seem to be involved with the regulation of gene expression [14].

Although still in the initial stages of functional understanding, many lncRNAs have been associated with major human diseases such as cancer [15], cardiovascular disease [16], and diabetes [17]. In the brain, several lncRNAs play a major role in its development and function [18], as well as in the pathogenesis of psychiatric disorders [11,19]. Recent research shows important involvement of lncRNAs in a variety of diseases including neurodegenerative disorders, such as Parkinson’s disease, Alzheimer’s disease, spinocerebellar ataxia, Huntington’s disease, and psychiatric disorders such as schizophrenia and bipolar disorder [20]. Additionally, over 50 candidate lncRNA genes were reported in a study connecting the noncoding human genome with intellectual disability, approximately half of which were further shown to be involved with synaptic transmission, neurogenesis, and nervous system development [21].

In a recent study, authors investigated transcribed RNA at three time points after simulating long-term potentiation to assess synaptic plasticity in rats and found many dynamically altered lncRNAs [22]. Human induced pluripotent stem cell (iPSC)-derived neurons were reported as a robust model for studying synaptic function [23]. Here, we investigate the shift in gene expression after iPSC-derived neurons were treated with potassium chloride (KCl), mimicking neuronal activation. Identifying differentially expressed genes in response to neural activation is important, as such events are essential in processes such as learning and memory formation. Indeed, disorders such as schizophrenia and autism are widely considered disorders of neuronal transmission [24].

## 2. Materials and Methods 

### 2.1. Generation and Differentiation of Induced Pluripotent Stem Cells

We sequenced total RNA content of hiPSC-derived neurons, both untreated and activated with 50 mM of KCl for 3 h. The studies were performed on de-identified human samples obtained for broadly consented scientific research. Details for the generation, activation, and sequencing of hiPSC-derived neurons were previously reported [25]. Briefly, human fibroblasts from apparently healthy individuals with normal psychiatric evaluations were obtained from Coriell collections GM04506 (female, 22 years of age), AG09319 (female, 24 years of age), and AG09429 (female, 25 years of age). Cells were cultured in human fibroblast (HF) cell medium and reprogrammed using lentiviruses expressing the transcription factor OCT4 and packaged in 293T human embryonic kidney (HEK) cells transfected with polyethylenimine. Human fibroblasts were infected daily for 5 days with tetracycline-inducible lentiviruses, and further transduced and split onto mouse embryonic fibroblasts. Cells were switched to human embryonic stem cells (HUES) medium supplemented with doxycycline for the first 21–28 days of reprogramming. Resulting embryoid bodies were transferred to non-adherent plates, kept in suspension in N2 medium for seven days, and plated onto polyornithine/laminin-coated plates. Rosettes were manually dissected and cultured in neural progenitor cells (NPCs) medium with laminin and fibroblast growth factor 2 (FGF2); the emerging NPCs were maintained at high density in NPC medium on polyornithine/laminin-coated plates and split regularly. For neural differentiation, NPCs were dissociated and plated at low density in neural differentiation medium onto polyornithine/laminin-coated plates. The hiPSC-derived neurons were differentiated for 1–3 months in fetal bovine serum (FBS)-supplemented co-culture with cerebellar astrocytes to improve conditions for in vitro synapse maturation.

The RNA-Sequencing (RNA-Seq) results were submitted to the sequence read archive (SRA) database and can be found under the accession numbers SAMN08095602, SAMN08095604, SAMN08095606 (controls) and SAMN08095601, SAMN08095603, SAMN08095605 (activated).

### 2.2. Neuronal Activation and RNA Sequencing

**Figure 1:**
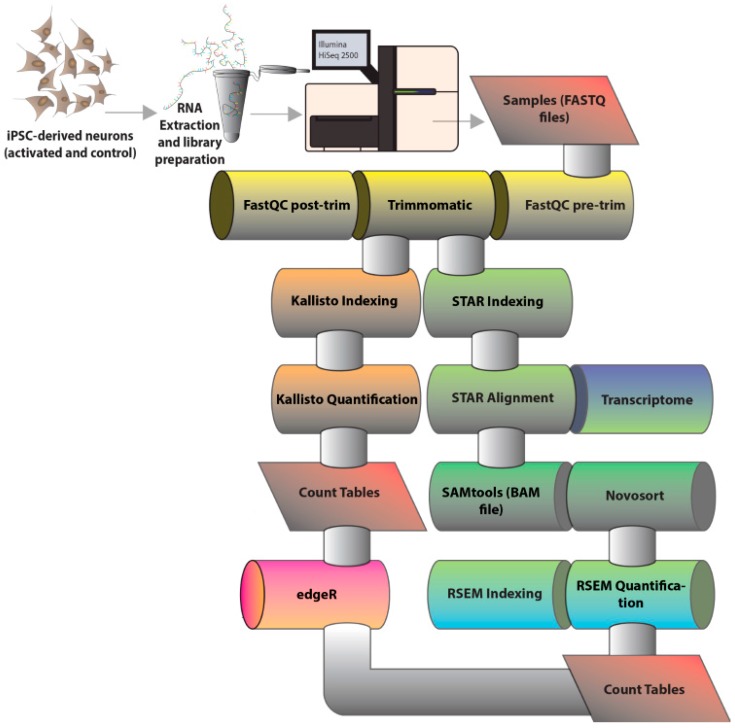
Bioinformatics pipeline for RNA-Sequencing (RNA-Seq) analysis. The figure schematically shows the steps for RNA-Seq analysis. After paired-end reads were generated by sequencing total RNA from human induced pluripotent stem cells (iPSC)-derived neurons using the Illumina HiSeq 2500 platform (Illumina, San Diego, CA, USA) and delivered as FastQ files. Bioinformatics tools were used to perform quality control (FastQC, version 0.11.5) [28] and adaptor trimming (Trimmomatic, version 0.36 [29]). From this point, reads were submitted to two parallel methods of transcript abundance estimation: an alignment-independent method where reads are simultaneously pseudoaligned and counted by Kallisto (version 0.43.0) [30]; and an alignment-based strategy where reads are first aligned to the reference transcriptome with Spliced Transcripts Alignment to a Reference (STAR)and then counted with RNA-Seq by Expectation Maximization (RSEM) (hereby the STAR+RSEM method) (versions 2.5.2 and 1.2.30, respectively) [31,32]. Both strategies generate count tables which are further normalized and can be assessed with EdgeR (R version 3.4.0) [33] for differential expression analysis.

Neurons were treated with 50 mM KCl (or phosphate-buffered saline (PBS) vehicle control) for the final three hours prior to harvest. KCl was dissolved in PBS as previously described [26], and, in all experiments, an equivalent volume of PBS was used as a vehicle control. The effects of this KCl dosage for treatment were previously investigated in molecular [26] and neurotransmitter release [27] studies. A 500 ng of ribosomal RNA (rRNA)-depleted total RNA was used as input material for library preparation, performed with the TruSeq Stranded Total RNA Kit (Illumina, San Diego, CA, USA), according to manufacturer-defined protocol. The resulting libraries were sequenced in the RapidRun V2 mode of Illumina HiSeq 2500 (Illumina) platform to generate 20–100 nt-long paired-end reads that were further analysed through a Bioinformatics pipeline (Figure 1).

### 2.3. Bioinformatics Analysis of RNA Sequencing Results

Each condition (control and activated with KCl) was sequenced in triplicate to allow statistical significance of subsequent analyses. Sequencing results were assessed for read length, per-base and per-sequence quality, GC content, and overall read composition with FastQC (version 0.11.5) [28]. Adaptor and other possible contaminants were trimmed with Trimmomatic (version 0.36) [29]. Reads were scanned with a 4-base wide sliding window and trimmed when the average quality per base was lower than 20 and only reads above our defined size cutoff of 40 nt were kept in the dataset. Two different strategies were chosen for read count to estimate transcript abundance, one using Kallisto (version 0.43.0) [30] and another using STAR+RSEM (versions 2.5.2 and 1.2.30, respectively) [31,32]. Kallisto is an alignment-free method, which does not need reads to be mapped to the genome or transcriptome, but instead relies on pseudoalignments to generate read counts based on k-mers (which we defined as 31 nucleotides in length). Reads were counted for transcript quantification using default parameters and a number of bootstrap samples of 100. STAR+RSEM, on the other hand, are two separate tools integrated into our pipeline to first align all reads to the reference transcriptome and further count reads based on the resulting alignment and provided annotation. Resulting alignment files were treated using Samtools (version 1.3) [34] and sorted with Novosort (version 1.2.30) [35] as an intermediate step. Regardless of the method, estimated read counts were submitted to EdgeR (R version 3.4.0) [33] for differential expression analyses, which include normalization and statistical tests of significance. A minimum of 5 counts per million was set as limit for transcript expression, with transcripts below this threshold being regarded as false-positives or transcriptional leakage. Normalized counts for all the remaining transcripts were contrasted between control and activated cell samples to reveal the set of differentially expressed transcripts, likely contributing to or being regulated by neuronal activation. Differential expression was reported, with limits being a p-value of less than 0.001 and a false discovery rate of less than 0.01. The sets of differentially expressed transcripts, resulting from both the alignment-free and alignment-based methodologies, were compared, and only the overlapping transcripts were used for subsequent analyses. Genes of interest were validated for activity-treated alterations using quantitative polymerase chain reaction(qPCR). Briefly, 500 ng of total RNA was used for complementary (cDNA) synthesis, and each cDNA sample was amplified in triplicate using SYBR Green PCR Master Mix (ThermoFisher Scientific, Waltham, MA, USA). For this experiment, two additional cell lines were included: Coriell collection GM03440 (male, 20 years) and GM03651 (female, 25 years).

### 2.4. Functional Analyses of Differentially Expressed Genes 

Functional analyses of differentially expressed genes were carried out using two different approaches. First, we used tools available at the Mississippi State University AgBase portal (version 2.0) [36] to assign ontologies to the transcripts of interest. GoAnna annotates genes by transferring ontologies based on sequence homology, and GoSlim further summarizes the results by providing a high-level annotation to analyzed genes. Basic local alignment search tool Protein (BLASTP) was used as the underlying algorithm, and search parameters were an e-value cutoff of 10 × 10^−50^, with blocks substitution matrix 62 (BLOSUM62), a minimum of 80% sequence identity plus 75% coverage, and default word size and gap penalty values. Significance of results was assessed using Fisher’s exact test to determine the respective *p*-value for each class when compared with the entire set of human proteins. In parallel, we used Bioconductor FGNet (version 3.6) [37] to build and enable visualization of functional gene networks, generated based on the functional enrichment analyses performed with the inbuilt GeneTerm Linker. 

## 3. Results and Discussion

### 3.1. Overall Quality of RNA-Sequencing Data

The RNA-Seq experiments generated over 75 million reads in total for control and over 60 million reads in total for activated neurons. Although we did not further investigate this discrepancy, the difference may be explained by a more specialized gene expression in activated neurons against a more scattered expression in control cells. The mode for overall read quality in the Phred scale (which is logarithmically correlated to the error probability during base-calling) is of 38 for forward reads (~1.6 errors in every 10,000 bases, or an accuracy of 99.98%) and 36 for reverse reads (~2.5 errors in every 10,000 bases, or an accuracy of 99.97%). Since a Phred score of 36 still represents very high accuracy, this small decrease in quality should not affect our analyses. Furthermore, although not properly discussed in the literature, it is generally observed that reverse reads tend to have lower quality values. In our case, it is due to lower quality scores at the end of the reads, which is expected, since errors tend to accumulate throughout the sequencing cycles and gradually decrease quality. Overall, the median Phred score is within the range considered for very good quality calls across all positions for both forward and reverse reads. In summary, our sequencing results were of very high overall quality and allow us to retrieve reliable information.

### 3.2. Differentially Expressed Transcripts

Using the alignment-based method for read counting, performed with STAR+RSEM, we found 853 transcripts to be differentially expressed (Table S1, 426 less expressed after neuronal activation and 427 more expressed after activation compared with inactive controls), coming from 798 different genes. From these, 14 are not protein-coding, with 7 transcripts downregulated upon activation and 7 upregulated upon activation (3 of which are intergenic lncRNAs). The alignment-free method of transcript abundance estimation using Kallisto reported 803 transcripts (Table S1, 394 downregulated and 409 transcripts upregulated after neuronal activation) from 751 genes, being 29 not protein-coding (15 downregulated upon activation and 14 upregulated upon activation).

**Figure 2:**
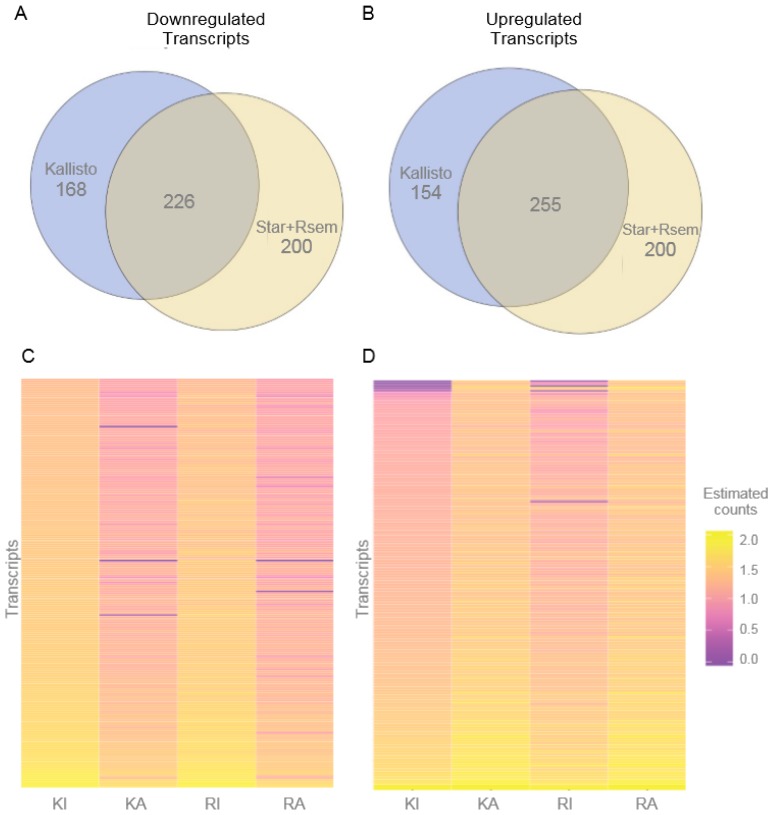
Differentially expressed genes between potassium chloride (KCl)-activated and control iPSC-derived neurons. Differentially expressed genes were identified using the alignment-free method implemented in Kallisto, and the alignment-based combined approach of STAR+RSEM. (**A**) Venn diagram showing genes with decreased expression in KCl-activated neurons were identified by each strategy and compared, revealing an intersection of 226 transcripts that were characterized as the valid set of downregulated transcripts. (**B**) Genes with increased expression in KCl-activated neurons were identified by each strategy and compared, revealing an intersection of 255 transcripts that were characterized as the valid set of upregulated transcripts. (**C**) Expression of downregulated genes (226 genes) and (**D**) expression of upregulated genes (255 genes) visualized as a heatmap where the color of each cell represents estimated and normalized read counts from purple to orange (i.e., low to high expression, in a logarithmic scale). KI: control sample assessed by Kallisto; KA: activated neurons sample assessed by Kallisto; RI: control sample with read counts from RSEM; RA: activated neurons sample with read counts from RSEM.

For each expression pattern (downregulated after activation or upregulated after activation), most the transcripts are consistently designated and differentially expressed by both methodologies, with 255 reported by both methods as upregulated and 226 as downregulated (both these overlaps have *p*-values of virtually 0) (Figure 2). It should be noted that because we only investigate one-time point (3 h) post KCl activation and not a time course, we are unsure of the dynamics of each transcript with respect to their peak change, onset, and duration. The fact that most transcripts are assigned the same behavior using different methods, and also that the number of transcripts in each group is similar regardless of the method, is suggestive of a robust result. Additionally, by using two different approaches we are likely to minimize any potential bias introduced by each tool. 

Using all (>0) estimate counts reported by Kallisto and STAR+RSEM, we calculated the Pearson (linear) and Spearman (ranking) correlations between results from both methods. We found these to be of 0.82 and 0.97, respectively (Figure S1). This high correlation, together with the fact that most transcripts were characterized in both methods by the same trend (upregulated/unaffected/downregulated) by neuronal activation—Figure 2 and Figure S2) support a robust qualitative result.

### 3.3. Functional Analysis of Differentially Expressed Transcripts

**Figure 3:**
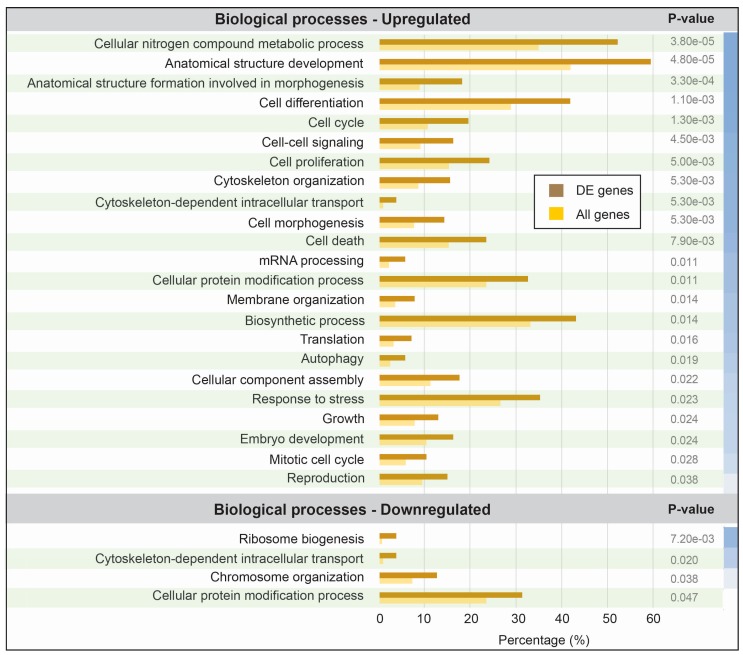
Gene ontology (GO) enrichment of differentially expressed transcripts between KCl-activated and control iPSC-derived neurons. The set of proteins derived from consistent differentially expressed transcripts was submitted to a gene ontology assignment protocol using tools from the AgBase portal (version 2.0) [25]. Results for the biological processes output were compared with the biological processes output for the entire set of annotated human proteins. Each broad class was quantitatively assessed to compare the percentage of entries from differentially expressed transcripts (brown) and the percentage of entries from the entire human proteome (yellow). A *p*-value was calculated for each class and the significantly overrepresented classes are shown in this figure. The blue color scale on the right represents the relative strength of each *p*-value, although all are below the significance cutoff of 0.05. DE: differentially expressed.

A total of 481 transcripts were consistently designated as being differentially expressed upon neuronal activation, using the two different bioinformatics methods (Figure 2). To investigate the functional impact of the protein-coding transcripts, we assigned ontology terms to each possible transcript and grouped them in different granularities by broad categories with umbrella terms. We further investigated the statistical significance of the abundance (proportional number of transcripts it comprises) per category, in contrast to the entire set of all human protein-coding genes. In total, three categories were found to be significantly (*p*-value < 0.05) over-represented and one to be significantly under-represented within the transcripts repressed by neuronal activation, while 24 were over-represented within transcripts induced by neuronal activation (Figure 3). These numbers alone may indicate that most of the impact of KCl activation comes from increasing the expression of transcripts from specific functional categories, in a regulated pattern.

The set of functions significantly associated with transcriptional upregulation following activation suggest the neurons are mainly altering their proliferation rates and differentiation state. Biological processes involved in these contexts include pathways of cell differentiation, cell cycle, cell proliferation, cell morphogenesis, and cell death. These categories also include a high number of differentially expressed genes, always above average, corroborating their responsiveness to neuronal activation. Interestingly, activation with KCl was previously reported to influence stem cell proliferation in the brain, thus complying with our findings [38]. It is also known that neuroblasts develop a mature neuronal phenotype in the presence of 25 mM KCl, while, in contrast, neurons undergo KCl withdrawal-induced apoptosis when KCl concentrations are lowered to 5 mM [39]. Long-term treatment with 25 mM KCl (~2 days exposure), on the other hand, resulted in a selective loss of cells committed to neuronal fate by both decreasing the rate of cell proliferation and increasing the rate of cell death [40]. In summary, there are multiple evidences of an interplay between neuronal activation via KCl exposure and the balance between cell proliferation and cell death.

**Figure 4:**
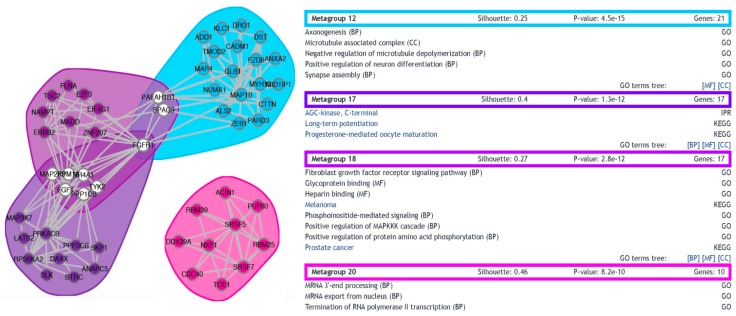
Functional network of differentially expressed transcripts. A functional network was built with functional gene networks (FGNet) (version 3.6) [37] based on the set of differentially expressed transcripts. The figure shows a filtered version of the full network, in which only metagroups of silhouette size greater than 0.2 are represented. On the left, the four surviving metagroups are graphically described, clustered by functional relationship in color-delimited areas. Genes are represented by circles and the connections between them by lines. White circles depict genes that were assigned to multiple metagroups. The number of genes, silhouette size, and *p*-value are also shown for all groups, as well as the functional categories they include (BP: Biological processes; MF: Molecular functions; CC: Cellular components).

To complement the ontology analysis, a functional network for the differentially expressed genes was generated, depicting protein-protein interactions and functional clusterization. In total, 20 metagroups were generated arising from the clustering of 273 genes (Figure S3). Three metagroups fail to produce a positive silhouette width (a negative silhouette width usually signals groups of genes with low functional coherence) and were filtered out of the original network. When a more stringent cutoff of 0.2 was applied as minimum silhouette size, four metagroups survived (Figure 4). Interestingly, the two most populated (and most significant, with lowest *p*-values) amongst these four groups are Metagroup 12 (21 genes associated with categories involved with synapse assembly, axonogenesis, microtubule dynamics, and neuron differentiation) and Metagroup 17 (17 genes associated with categories including long-term potentiation).

### 3.4. Differentially Expressed Non-Coding RNAs

**Figure 5:**
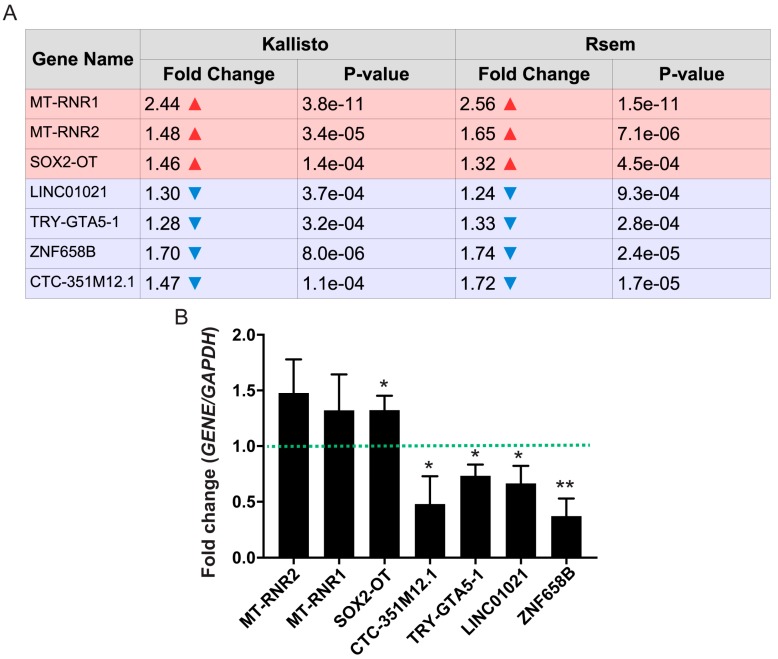
Differential expression of non-coding RNAs (ncRNA) genes between KCl-activated and control iPSC-derived neurons. Differentially expressed ncRNA genes were classified as either upregulated (*MT-RNR1*, *MT-RNR2*, and *SOX2-OT*) or downregulated (*LINC01021*, *TRY-GTA5-1*, *ZNF658B*, and *CTC-351M12.1*) by neuronal activation. (**A**) Fold-changes calculated based on RNA-Seq data are shown in the table based on both Kallisto-generated and RSEM-generated read counts. In red are the genes that are upregulated in response to neuronal activation, with their respective fold-change (in logarithmic scale) and *p*-values. The same is shown in blue for downregulated genes. (**B**) Quantitative polymerase chain reaction (qPCR) for all significantly regulated, activity-dependent ncRNA genes predicted by the RNA-Seq results (*n* = 5; * *p*-value < 0.05; ** *p*-value < 0.01; one-way analysis of variance (ANOVA) with post-hoc Tukey’s multiple comparison test).

From the 481 transcripts shown to be differentially expressed, 7 were annotated as non protein-coding genes, with 3 upregulated in activated cells and 4 downregulated, compared with controls. We explored the functional annotations of these ncRNAs to contextualize their differential expression after activation of neurons. The ncRNAs that are upregulated upon activation are *MT-RNR1*, *MT-RNR2*, and *SOX2-OT*, while those downregulated are *LINC01021*, *TRY-GTA5-1*, *ZNF658B*, and *CTC-351M12.1* (Figure 5A). These genes were also assessed by qPCR to confirm their differential expression, and results are in agreement with observations from the RNA-Seq (Figure 5B and Table S2) for fold change relative to *GAPDH* expression (*GAPDH* was shown by RNA-Seq to not alter its expression in response to neuronal activation with KCl).

*MT-RNR1* and *MT-RNR2* are mitochondrially encoded 12S and 16S RNAs, respectively. Interestingly, *MT-RNR2* (ENST00000387347) is one of the very few characterized examples of a bifunctional ncRNA, producing a protein called humanin, which was described as a neurosurvival factor in brains of Alzheimer’s disease patients [41]. Further studies propose that humanin may protect cells from apoptosis induced by amyloid β 1–43 by binding to Bcl2-associated X protein (BAX), an apoptosis-inducing factor, and preventing its translocation to the mitochondria [42,43,44]. Recently, *MT-RNR1* (ENST00000389680) was also shown to generate a peptide (MOTS-C) that regulates sensitivity to insulin and metabolic homeostasis, protecting against insulin resistance triggered by ageing or diet [45]. These two peptide-encoding ribosomal RNA genes suggest that mitochondria may actively influence cell death and metabolism via peptides encoded by their ncRNAs and may be triggered by neuronal activity.

The lncRNA locus *SOX2-OT* produces overlapping transcripts to the *SOX2* gene, an important regulator of neurogenesis. Accordingly, *SOX2-OT* expression seems to be confined to the brain, with a very low but detectable expression in testis [46]. A recent study using single-cell RNA sequencing determined *SOX2-OT* to be one of the few highly expressed lncRNAs in the human neocortex [47]. The expression patterns of *SOX2-OT* are highly regulated throughout vertebrate development [48] and have been detected in mouse adult neurogenic regions [49]. *SOX2-OT* has also been observed to be differentially expressed in many types of cancer [50,51,52,53] and was strongly associated with eating disorders in bipolar patients [54].

*LINC01021* (ENST00000512067) is a long intergenic non-protein coding RNA (lincRNA), also known as p53 upregulated regulator of p53 levels (*PURPL*). *PURPL* suppresses basal p53 levels, leading to tumorigenicity [55]. Expression levels for LINC01021 (calculated herein) are higher in control samples, before KCl-mediated activation. High levels of this lincRNA suppress p53, avoiding cell cycle arrest and apoptosis. *PURPL* expression in human neural progenitor cells was also shown to be controlled by *CHD8*, a leading autism spectrum disorder candidate gene [56], which we observe to follow *LINC01201* expression pattern. Another gene significantly downregulated upon activation is *TRY-GTA5-1* (ENST00000517961), a transfer RNA for tyrosine with reported high expression in testis [46]. The zinc finger protein 658 (ZNF658) is a transcription factor of the C2H2-type zinc-finger protein family. *ZNF658B* (ENST00000602828) is a pseudogene, which our results suggest to be significantly downregulated upon neuronal activation. *ZNF658* was recently shown to mediate the cell transcriptional response in response to zinc, especially regarding ribosome biogenesis [57]. Lastly, *CTC-351M12.1* (ENST0000062483) is a currently uncategorized ncRNA, still to be experimentally confirmed, and our results may be one of the few to show its regulated expression. This gene is listed in a database of genes involved in angiogenesis but has yet to be experimentally validated [58].

### 3.5. Human-Specific Differentially Expressed Genes

Young genes (i.e., genes that have recently evolved, mainly after the divergence between hominids and other primates) are strikingly often recruited to the brain, more specifically, the neocortex [59]. For this reason, we decided to investigate genes that have been previously associated with brain function and/or development and have recently evolved or significantly changed. The list presented below is intended to represent an overview of such interplay between new genes and brain evolution, and these examples are not comprehensive.

Genes with human-specific features that are differentially expressed upon neuronal activation include *CAPN1*, which is required for immune function [60]; *TLK2*, actively involved in mitosis [61,62]; *AFF3*, a transcription factor involved in cellular migration and cortical development that when deficient leads to intellectual disability [63]; *KIAA0319L*, which may be involved in language dysfunction [64]; and *RBM39*, an RNA-binding protein that plays a role in messenger RNA (mRNA) splicing [65]. Only *CAPN1* is upregulated upon neuronal activation with KCl, and all additional four genes are downregulated (Table S3). *RBM39* is the most downregulated transcript of all (not only amongst human-specific transcripts), shifting from very high expression levels before activation to being virtually absent after activation. 

While *KIAA0319* is involved in neural migration and outgrowth and is associated with dyslexia susceptibility [66], its structural homologue *KIAA0319L* is an adeno-associated virus receptor that mediates viral infection [67]. It is interesting that two structural homologues have neurological or immunological roles, maybe as a result of both systems being highly adaptable and evolutionarily and developmentally interlinked [68,69]. *TLK2*, another differentially expressed gene is required for placental development and, while coupling with *TLK1*, supports genomic stability in mice by recovery of damaged DNA in preparation for mitosis [70,71]. When coupled with *ASF1*, *TLK2* restores chromatin structure after DNA damage and assists further progression through the cell cycle [71].

### 3.6. Highly Expressed Transcripts

**Figure 6:**
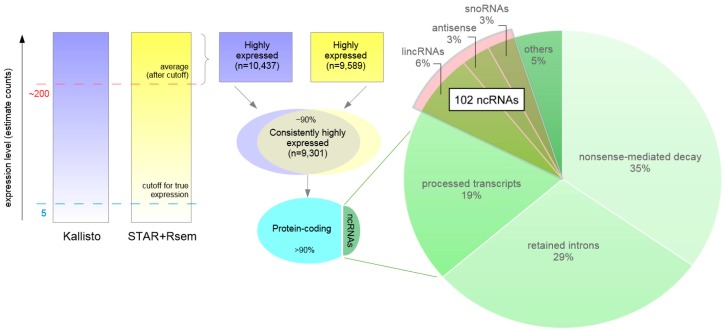
Transcripts that are highly expressed in activated neurons. Strategy used to assess transcripts with high expression in activated neurons. Transcript abundance is estimated by Kallisto (purple) and STAR+RSEM (yellow). The color intensity represents expression level in estimated counts. A lower limit for expression confidence was established at 5 counts (blue dashed line). To be considered highly expressed the average number of estimated counts was above approximately 200 (red dashed line). There were approximately 10,000 highly expressed transcripts defined by each method, more than 9000 of which were consistently reported by both methods. From these, approximately 10% were ncRNAs, a total of more than 800 transcripts. The pie chart represents the distribution of these ncRNAs in their molecular classes and highlights the set of 102 highly expressed ncRNAs, described in more detail in the text, in which a higher degree of functional data is available. lincRNA: large intergenic non-coding RNA; snoRNA: small nucleolar RNA.

In addition to activation-dependent, differentially expressed transcripts, highly expressed transcripts, although not significantly affected by neuronal activation, may be of interest in our human neural system. Considering that the distribution of estimate counts is strongly positively skewed (the average values for both Kallisto and STAR+RSEM estimated counts are ~200, while the medians are set at ~25), we have defined transcripts with counts above average as highly expressed. This defined set comprises ~10,000 transcripts, which represents ~5% of the total number of transcripts. From the 10,437 highly expressed transcripts reported by Kallisto and the 9589 transcripts reported by STAR+RSEM, 9301 (nearly 90%) are reported by both methods, suggesting a high consistency (Figure 6).

### 3.7. Highly Expressed Protein-Coding Transcripts Involved in Brain Function, Development, and Evolution

As a proof of principle, we briefly describe some highly expressed protein-coding genes to highlight the idea that high expression is related to a high degree of functionality. Furthermore, we only focus on genes with critical human neural function. For example, the highly expressed abnormal spindle-like microcephaly associated (*ASPM*) gene displays accelerated sequence evolution after the split of humans and chimpanzees, controls brain development, and is one of the main genes that dictate brain size [72]. The highly expressed microcephalin (*MCPH1*) gene also regulates brain size, being essential for normal cerebral cortical growth, which suggests critical roles for these two genes in the substantial increase in brain cortex size in humans [73]. Similarly, the evolution of the highly expressed CDK5 regulatory subunit associated protein 2 encoding gene (*CDK5RAP2*) is also strongly correlated with brain size, suggesting it carries out important functions in brain development [74]. While mice have only one copy of the cortical development gene SLIT-ROBO Rho guanosine-5'-triphosphatase (GTPase)-activating protein 2 (SRGAP2), the inversion and segmental duplication on human chromosome 1 created new human-specific paralogs, including *SRGAP2C* [74,75]. *SRGAP2C* (which is the only *SRGAP2* transcript highly expressed in our dataset) is of particular importance, since it was recently shown to slow the maturation of certain brain cells, trigger the development of a denser array of neuronal spines, and influence how neurons migrate in a developing brain [75]. In addition to *SRGAP2*, the inversion on chromosome 1 contains copy number increases of the developmental gene, *HYDIN*, and the neuroblastoma breakpoint family (NBPF), from which we observed *NBPF1* to be very highly expressed, and this gene is involved in human-specific development [76]. Finally, *SOX2* transcripts are amongst the 1% most highly expressed, and this protein is a classical promoter of neurogenesis [77]. There are many more examples in our dataset of highly expressed protein-coding genes that contain human-specific sequence and are involved in brain function (Box S1). However, our goal was to identify potential non-coding RNAs involved in human brain function, and these protein-coding examples support the idea that high expression may be relevant for increased confidence of functionality.

### 3.8. Highly Expressed ncRNAs

From our set of 9301 transcripts (originating from ~6800 genes), almost 10% are transcripts that do not seem to encode proteins (813, from 669 genes), including 8 of the top 10 most highly expressed transcripts. Interestingly, nearly 20 ncRNAs are contained in the top 10% of these highly expressed transcripts, including *MALAT1*, *MT-RNR1*, and *MT-RNR2* (which are upregulated upon neuronal activation, as previously discussed). Amongst these non protein-coding, highly expressed transcripts (Figure 6), most are either truncated transcription products that undergo nonsense-mediated decay (283), retained introns (234), or processed transcripts, which is any transcript lacking an open reading frame (154). The next most abundant categories (with a combined number of 102 genes) are long intervening noncoding RNAs (lincRNAs) (55), antisense transcripts (24), and small nucleolar RNAs (snoRNAs) (23), followed by subclasses of the former categories, or minor classes, each representing less than 10% of the 813 transcripts. Here we focus on the middle strata, the set of 102 transcripts that we believe are of particular interest due to the presence of some knowledge of functionality for these classes of RNA.

From the lincRNAs, *MALAT1* is highly expressed (with one isoform in the top 10 most expressed transcripts), which is a frequently reported lncRNA and was previously shown to promote neurite outgrowth, prevent cell death, and be important in the early stage of neuronal differentiation [78]. Also highly expressed was Gomafu, a lincRNA previously shown by our group to be involved with schizophrenia and respond to neuronal activation [79]. Further, rhabdomyosarcoma 2-associated transcript (RMST), is a highly expressed lncRNA and a crucial regulator of neurogenesis that physically interact with *SOX2* [80]. *NEAT1*, an essential component of nuclear paraspeckles previously investigated by our group and shown to respond to neuronal activity [81], is also highly expressed. Other highly expressed transcripts include the uncharacterized *LINC00461*, with reported brain-specific expression; *LINC00998*, implicated in major depression disorder susceptibility [82]; *EPB41L4A*, a candidate regulator of anxiety disorders [83]; *C1orf132*, a gene for which methylation levels accurately correlate with chronological age [84,85]; and *BAIAP2*, which was previously associated with memory maintenance and linked to attention deficit and hyperactivity disorder [86,87]. 

Highly expressed antisense transcripts include *SNHG14*, previously shown to promote microglia activation [88] that may therefore also be involved with neuronal activation; antisense transcripts to a proteasome component and a deubiquitinating enzyme (*PSMA3-AS1* and *OTUD6B-AS1*, respectively), therefore implicating protein degradation as a possible mechanism involved with neuronal activation; *OIP5-AS1* (also known as cyrano), a lncRNA with reported high abundance in mammals that regulates cell proliferation and maintenance of self-renewal in stem-cells [89,90]; and *KCNQ1OT1* (also known as *LIT1*), a *DNMT1*-interacting ncRNA that mediates transcriptional silencing [91,92]. These examples uncover new potential genes involved in human neuronal function and future mechanistic work may determine their precise involvement.

## 4. Conclusions

The human brain is one of the most complex structures known. Its intricate architecture is still mysterious and this is partially due to the fact that human brains (especially healthy ones or those that carry rare diseases) are not available to study in the laboratory at a molecular level. The advent of iPSC technology and the capacity of iPSCs to be transformed into dynamic neuronal networks is an innovative strategy to help overcome this gap. In the past few years, neurons generated from iPSCs have fueled the study of neurologic and psychiatric diseases, as well as contributing to the overall understanding of the normal brain [93,94]. These cellular constructs provide a platform for the study of the human brain, which is now unprecedentedly accessible. Here we have used KCl-treated hiPSC-derived neurons to shed light on transcripts that may participate in neuronal activity. Our RNA-Seq results uncover multiple lncRNAs as dynamically altered in response to KCl-mediated neuronal activation and implicate pathways that drive cellular proliferation, differentiation, and the emergence of specialized morphological features (such as dendrites, axons, and nuclear paraspeckles). Many lncRNAs are present in the list of highly expressed genes in activated neurons, and some respond to activation with KCl. These RNAs are likely to function alongside proteins in orchestrated molecular networks that ultimately create what we define as higher intelligence.

## Supplementary Materials

The following are available online at www.mdpi.com/2073-4425/8/12/401/s1. Figure S1: Correlation between the two methodological approaches for transcript abundance estimation; Figure S2: Intersection between methods in terms of transcript expression; Figure S3: Full functional network for differentially expressed genes between controls and KCl-activated neurons; Box S1: Other examples of highly expressed transcripts with previously reported roles in the brain; Table S1: Differentially expressed transcripts; Table S2: qPCR primers; Table S3: Metrics for expression of genes/transcript of interest.
